# Outcomes of infants with birthweights less than 501 g compared to infants weighing 501–750 g at a center utilizing first intention high frequency jet ventilation

**DOI:** 10.3389/fped.2024.1392079

**Published:** 2024-09-09

**Authors:** Timothy G. Elgin, Jennifer N. Berger, Dinushan C. Kaluarachchi, John M. Dagle, Brady Thomas, Tarah T. Colaizy, Jonathan M. Klein

**Affiliations:** ^1^ Department of Pediatrics, University of Wisconsin School of Medicine and Public Health, Madison, WI, United States; ^2^ Children’s Minnesota, Neonatal Medicine, Minneapolis, MN, United States; ^3^ Stead Family Department of Pediatrics, University of Iowa, Iowa City, IA, United States

**Keywords:** neonatalogy, HFJV - high-frequency jet ventilation, high frequency, survival, nutrition

## Abstract

**Background:**

Data on clinical outcomes of infants with birthweights less than 501 g (ELBW^<501^) are limited.

**Objective:**

To evaluate management strategies and clinical outcomes of ELBW^<501^infants compared to infants weighing 501–750 g (ELBW^501–750^).

**Methods:**

A retrospective study of all ELBW^<501^ and ELBW^501–750^ infants born between 2012 and 2022 at a center utilizing first intention high frequency jet ventilation was performed. Patient characteristics, clinical and outcome data were compared between the two groups.

**Results:**

A total of 358 infants (92 ELBW^<501^ infants and 266 ELBW^501–750^) were included. The survival rate for the ELBW^<501^ group was 60.9% compared to 86.5% for ELBW^501–750^. ELBW^<501^ infants required more frequent use of 2.0 mm endotracheal tubes, required higher FiO_2_ and longer duration of mechanical ventilation. Compared to ELBW^501–750^ group, the ELBW^<501^ group were more likely to be SGA (68.2% vs. 16.5%) and more premature (23.2 vs. 24.3 weeks) with lower survival, longer length of stay, higher incidence of ROP and lower weight at discharge but comparable rates of IVH, grade 3 BPD, discharged on supplemental oxygen, and tracheostomy.

**Conclusion:**

ELBW^<501^ infants are at risk for significant morbidity and mortality. However, with specialized obstetric and neonatal care, survival rates of 60% are possible with respiratory outcomes comparable to ELBW^501–750^ infants. However, the increased risk of severe ROP for ELBW^<501^ requiring either surgical or medical intervention is concerning and warrants optimal surveillance.

## Introduction

The rates of survival for infants born at lower gestational ages and birthweights have been increasing over the past 2 decades. As survival of extremely low birthweight infants (ELBW, birthweight <1,000 g) has increased worldwide, the rates of infants with birthweights of <501 g who are surviving has also increased (1). Infants born with birthweights <501 g are distinctly different in terms of survival, morbidity, and long-term prognosis compared to VLBW and ELBW infants. While gestational age is often at the forefront of discussions regarding survivability and long-term outcomes, it is important to remember the role that birthweight plays in survival. Unfortunately, there is limited data on the outcomes and management strategies for this unique patient population of infants with birthweights under 501 g. The University of Iowa has had success managing infants born at the limits of viability including follow up data demonstrating a low risk of severe neurodevelopmental impairment at corrected age of 18–22 months for infants born at 22–25 weeks gestation with a standardized approach to the management of nutrition, mechanical ventilation, and hemodynamics (2–5). In the current study, we describe and compare the mortality and morbidity of inborn infants born weighing less than 501 g to infants weighing between 501 and 750 g at a single center over eleven years managed with a consistent respiratory strategy focused on first intention high frequency jet ventilation. We used our data submitted to the Vermont Oxford Network (VON) to compare mortality and morbidity between ELBW^<501^ and ELBW^501–750^ infants. From a separate database containing information from July 1, 2012 through June 30, 2017, we abstracted data comparing high frequency jet ventilator characteristics between these same two groups (6).

## Methods

A retrospective cohort analysis of infants born at the University of Iowa between January 1st, 2012, and December 31st, 2022, with a birthweight ≤750 g. Infants were grouped into two birthweight categories, less than 501 g referred to as ELBW^<501^ and infants weighing between 501 and 750 g, referred to as ELBW^501–750^. Infants were excluded if they were out born, or parents declined active resuscitation at the time of birth. Neonatal characteristics, diagnoses and outcome data were compiled from Vermont Oxford Network (VON) database. All VON data were site-specific, and data were collected in accordance with the data definitions governed by VON. VON is a non-profit collaboration comprised of NICUs across the world. Racial demographic data per VON definitions.

Gestational age was determined by standard clinical guidelines using last menstrual period, prenatal ultrasonography, or physical examination. Birthweight was measured using the electronic scales on the infant beds on admission to the NICU. Antenatal steroid therapy was defined as any corticosteroid administered to the pregnant mother prior to delivery for the purpose of enhancing fetal lung maturity. Infants were classified as being small for gestational age (SGA) when the birthweight was less than the 10th percentile for gestational age and sex according to the Fenton Growth chart (7). Extreme length of stay was determined per VON definition and reflects whether an infant's total hospital stay is greater than the 95th percentile for predicted value. Bronchopulmonary dysplasia (BPD) was defined using the classification system proposed by Jensen et al. based on respiratory support required at 36 weeks postmenstrual age (PMA) (8). Grade 1 BPD is defined as oxygen by nasal cannula at ≤2 LPM flow, Grade 2 as nasal cannula flow >2 LPM flow or noninvasive positive pressure and Grade 3 is represented by invasive mechanical ventilation via endotracheal tube (8). Intraventricular hemorrhage (IVH) grade was reported based on the Papile classification system on cranial ultrasound performed in all infants during the first week of life and at 36 weeks PMA (9). Periventricular leukomalacia (PVL) was defined as radiologist identified PVL on cranial ultrasound, CT or brain MRI. Retinopathy of prematurity (ROP) presence and stage was reported based on diagnosis from ophthalmologic exam by study institution's ophthalmology team, and severe ROP was defined using VON definitions (stage 3, 4, or 5) (10).

Necrotizing enterocolitis (NEC) was defined per the VON definition as present if diagnosed at surgery, at postmortem examination, or with clinical and diagnostic imaging using the following criteria: at least one of the following clinical signs present: bilious gastric aspirate or emesis, abdominal distension or discoloration, occult or gross blood in stool (no fissure) and at least one of the following diagnostic imaging findings present: pneumatosis intestinalis, hepato-biliary gas, or pneumoperitoneum (11, 12).

From a separate time restricted (July 1, 2012 through June 30, 2017) respiratory database (University of Iowa NICU registry, electronic health record systemic, Epic, as collected in RedCap), we abstracted data comparing high frequency jet ventilator and respiratory characteristics between the <501 and the 501–750 g birthweight cohorts. Respiratory data collected included mode of ventilation and settings, days on mechanical ventilation, age at first and final successful extubation attempt, age at weaning to low flow oxygen, and days on non-invasive respiratory support capable of delivering positive pressure including non-invasive ventilation. Non-invasive ventilation included neurally adjusted ventilatory assist (NAVA), nasal pharyngeal intermittent (6) mandatory ventilation (NP-IMV), nasal pharyngeal continuous positive pressure airway pressure (NP-CPAP), RAM Cannula, and high flow nasal cannula >2 LPM.

## Statistical analysis

Data were calculated using frequencies and percentages for categorical factors or with the median and interquartile range (IQR) for continuous characteristics. Demographic, survival, respiratory outcomes, ventilator characteristics and morbidity outcomes were compared between ELBW^<501^ and ELBW^501–750^ using Mann-Whitney tests for continuous variables and Chi Squared or Fisher's exact test for categorical variables. Differences were considered significant when the *p*-value was <0.05. No statistical adjustments were made for multiple testing. All analyses were performed using GraphPad Prism version 9.0.

## Results

**Figure 1:**
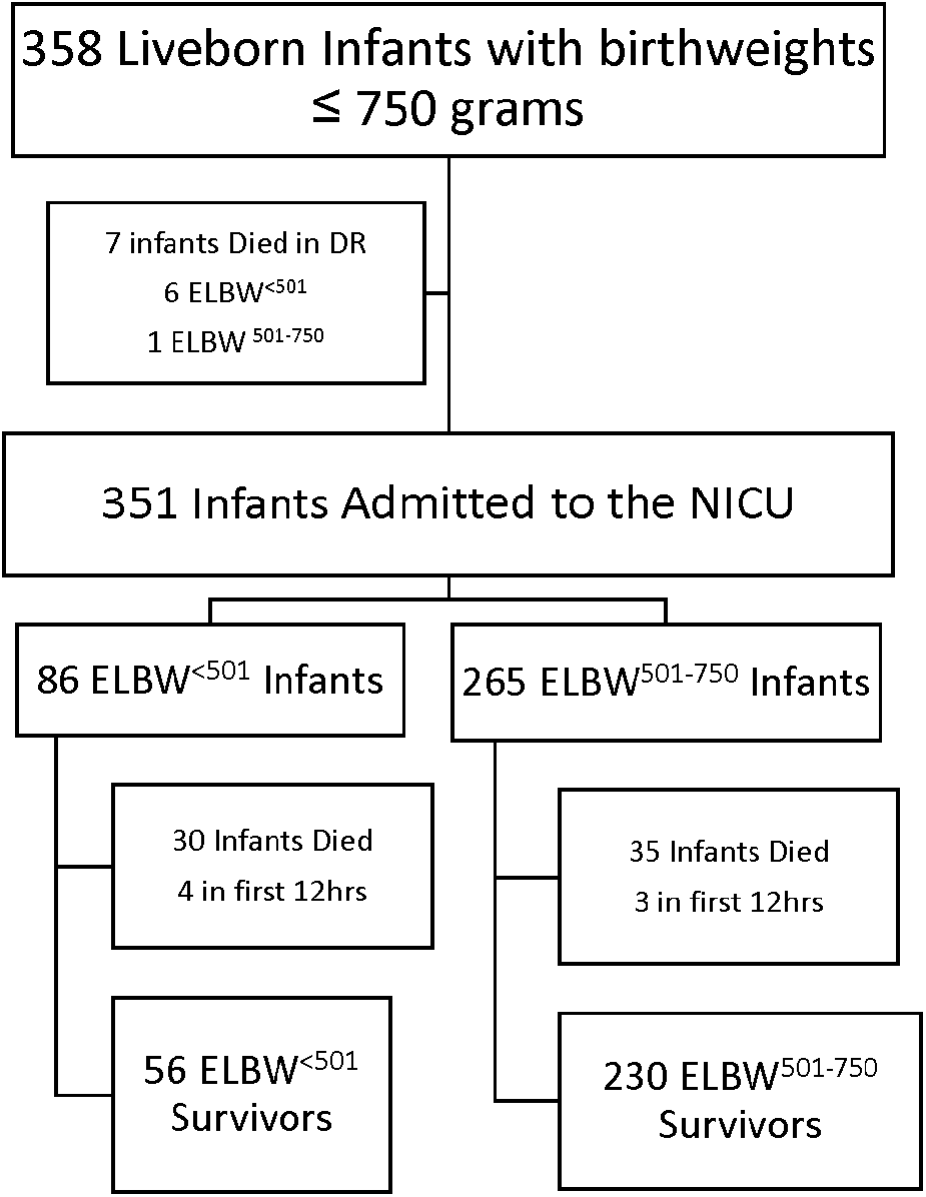
Consort diagram of infants born ≤750 g from 2012–2022.

A total of 358 infants with birthweights ≤750 g were born between January 1, 2012 and December 31, 2022 (Figure 1), 92 categorized as ELBW^<501^ and 266 as ELBW^501–750^. Demographics data are presented in Table 1. There were 7 infants who died in the delivery room, 6 in the ELBW^<501^ group and 1 in the ELBW^501–750^ group (*p* < 0.01). There were 351 infants who were admitted to the NICU. Of these infants, 86 were born weighing less than 501 g and 265 were born weighing between 501 and 750 g. Four total infants died within the first 12 h in the ELBW^<501^ group and 3 infants died within the first 12 h in the ELBW^501–750^ group (*p* = 0.04). ELBW^<501^ infants had a mean birthweight of 440 g vs. 636 g in the ELBW^501–750^ group (*p* < 0.01). ELBW^<501^ infants were born at significantly lower gestational ages (median 23.2 weeks vs. 24.3 weeks, *p* ≤ 0.01) and had a similar percentage of male infants (46.7% vs. 48.5%, *p* = 0.28) compared with the ELBW^501–750^ group. ELBW^<501^ infants were less likely to have received any antenatal steroids (91.3% vs. 97.4%, *p* = 0.01) and were more likely to be hypothermic on admission with an initial temperature between 32.0 and 35.9 degrees Celsius when compared to the ELBW^501–750^ group (54.2% vs. 34.9%, *p* < 0.01). Caesarian sections were less common in the ELBW^<501^ group then in the ELBW^501–750^ group (44.6% vs. 63.4%, *p* < 0.01). Diagnosis of maternal chorioamnionitis was not different and there were similar rates of multiple gestation pregnancies between the two groups.

**Table 1:** Demographic information of ELBW^<501^ and ELBW^501–750^ infants.

	ELBW^<501^ *N* = 92	ELBW^501–750^ *N* = 266	*p*-value
Birth weight g (mean, SD)	440, 44	636, 73	**<0**.**01**
Gestational age weeks (mean, SD)	23.2, 1.8	24.3, 1.7	**<0**.**01**
Male *n*, %	43 (46.70%)	49 (48.50%)	0.29
Received at least 1 dose of antenatal steroids *n*, %	84 (91.3%)	259 (97.4%)	**0**.**01**
Race/ethnicity			
Non-Hispanic White *n*, %	63 (68.5%)	174 (65.4%)	0.59
Non-Hispanic Black or African American *n*, %	15 (16.3%)	55 (20.7%)	0.36
Hispanic, *n*, %	9 (9.8%)	23 (8.6%)	0.74
Non-Hispanic Asian *n*, %	2 (2.2%)	9 (3.2%)	0.56
Other *n*, %	3 (3.3%)	5 (1.9%)	0.44
Cesarian section *n*, %	41 (44.6%)	166 (62.4%)	**<0**.**01**
Chorioamnionitis *n*, %	18 (19.6%)	55 (20.8%)	0.81
Multiple gestation *n*, %	27 (29.3%)	65 (24.4%)	0.35
Small for gestational age *n*, %	45 (68.2%)	43 (16.5%)	**<0**.**01**
Admit hypothermia (32.0–35.9°C) *n*, %	45 (54.2%)	90 (34.9%)	**<0**.**01**
Died within delivery room/operating room *n*, %	6 (6.5%)	1 (0.4%)	**<0**.**01**
Died within 12 h of life *n*, %	4 (4.7%)	3 (1.1%)	**0**.**04**

SD, standard deviation; ELBW, extremely low birth weight.

Bold values represent statistical significance.

Neonatal outcomes are presented in Table 2. ELBW^<501^ infant survival was significantly lower compared to the ELBW^501–750^ infants (60.9% vs. 86.5%, *p* < 0.01). Extreme length of stay was significantly more common in infants born less than 501 g (22.2% vs. 7.5%). The rate of any IVH between the groups did not reach significance, neither did the presence of severe IVH or rates of cystic periventricular leukomalacia. The diagnosis of NEC did not reach statistical significance between the two groups. PDA diagnosis rates were similar between the two groups, 73.3% and 72.5% (*p* = 0.88). Additionally, rates of early onset bacterial sepsis, late onset bacterial sepsis, and fungal sepsis were not significantly different between the groups. ELBW^<501^ infants were more likely to be diagnosed with any ROP (91.2%, vs. 68.2%, *p* < 0.01), and to have a higher incidence of severe ROP (28.1% vs. 10.3%, *p* < 0.01) compared to ELBW^501–750^ infants. Rates of anti-VEGF medication administration or laser therapy was significantly higher in ELBW^<501^ (15.1% vs. 5.1%, *p* < 0.01). Oxygen use at 36 weeks post menstrual age for ELBW^<501^ infants was 100%, and 95.2% in ELBW^501–750^ infants (*p* = 0.09). There was no difference in rates of grade 3 BPD (14% vs. 11%, *p* = 0.52) or home going oxygen (95.8% vs. 95.1%, *p* = 0.83) between the groups. There was no difference seen in rates of tracheostomy placement. More infants in the ELBW^<501^ group had weight <10%tile at time of discharge (35% vs. 20.3%).

**Table 2:** Neonatal outcomes between ELBW^<501^ and ELBW^501–750^ infants.

	ELBW^<501^	ELBW^501–750^	*p*-value
Survival *n*, %	56 (60.9%)	230 (86.5%)	**<0** **.** **01**
Extreme length of stay *n*, %	10 (22.2%)	17 (7.5%)	**<0**.**01**
Any IVH, *n*, %	22 (27.5%)	62 (24.2%)	0.55
Severe IVH (Grade III or IV) *n*, %	13 (16.3%)	25 (9.8%)	0.11
Cystic PVL *n*, %	1 (1.2%)	15 (5.8%)	0.09
PDA diagnosed *n*, %	63 (73.3%)	192 (72.5%)	0.88
NEC	5 (5.8%)	13 (4.9%)	0.74
Oxygen at 36 weeks, *n*, %	57 (100%)	216 (95%)	0.09
Stage 3 BPD, *n*, %	8 (14%)	25 (11%)	0.52
Oxygen at time of discharge, *n*, %	46 (95.8%)	194 (95.1%)	0.83
Any ROP, *n*, %	52 (91.2%)	159 (68.2%)	**<0**.**01**
Severe ROP, *n*, %	16 (28.1%)	24 (10.3%)	**<0**.**01**
Anti-VEGF medication, *n*, %	7 (8.1%)	6 (2.3%)	0.06
ROP laser therapy *n*, %	6 (7.0%)	7 (2.6)	0.06
Anti-VEGF or laser therapy *n*, %	13 (15.1%)	13 (5.1%)	**<0**.**01**
Early bacterial infection, *n*, %	4, (4.7%)	6 (2.3%)	0.24
Late bacterial infection, *n*, %	14 (18.9%)	44 (17.1)	0.71
Any fungal infection, *n*, %	1 (1.4%)	4 (1.6%)	0.9
Any post-natal steroids *n*, %	42 (48.8%)	115 (43.4%)	0.38
Any surfactant *n*, %	85 (92.4%)	255 (95.9%)	0.19
Tracheostomy *n*, %	5 (5.8%)	8 (3.0%)	0.23
Head circumference <10%tile at time of discharge *n*, %	14 (38.9%)	54 (28.3%)	0.2
Weight <10%tile at time of discharge *n*, %	14 (35%)	41 (20.3%)	**0**.**04**

IVH, intraventricular hemorrhage; HUS, head ultrasound; PVL, periventricular leukomalacia; PDA, patent ductus arteriosus; NEC, necrotizing enterocolitis; ROP, retinopathy of prematurity; BPD, bronchopulmonary dysplasia.

(*n* reflects total number of infants with documentation, surviving or eligible for inclusion within each category, and therefore may not add up total number of either ELBW^<501^ or ELBW^501–750^ infants).

Bold values represent statistical significance.

Respiratory and ventilator characteristics for the July 1st, 2012 through June 30th, 2017 cohort were obtained from a previous dataset and are presented in Table 3 (6). ELBW^<501^ infants were more likely to be initially intubated with a 2.0 mm ETT (89% vs. 32%, *p* < 0.01) whereas ELBW^501–750^ infants were intubated more often with a 2.5 mm ETT (66% vs. 11%, *p* < 0.01). Nearly all infants, regardless of group, were initially placed on the HFJV, consistent with unit policy (100% vs. 95%, *p* = 0.15). ELBW^<501^ infants were found to have a significantly higher oxygen need at 6 h of life (median 33% vs. 25%, *p* < 0.01), however no differences were seen in MAP, HFJV PIP, HFJV rate, or PCO2 at 6 h of life. At 7 days of life, ELBW^<501^ infants required significantly higher HFJV PIP than ELBW^501–750^ infants (median PIP 30 vs. 26, *p* < 0.01) but no difference was seen in FiO_2_, MAP, HFJV rate, or PCO2 at that time point. At extubation, there were no differences in MAP, HFJV PIP or rate, FiO_2_, or PCO2 between the two groups, however ELBW^<501^ infants' first trial of extubation occurred at a older age (day of life 56 vs. 44, *p* = 0.0007) and older PMA (31.6 vs. 30.57, *p* = 0.049) than ELBW^501–750^ infants. Additionally, successful extubation also occurred at a later day of life for ELBW^<501^ infants than ELBW^501–750^ infants (median 68.5 vs. 50.5, *p* < 0.01), though interestingly there was no difference in postmenstrual age at final extubation (33.36 vs. 32.12, *p* = 0.12). ELBW^<501^ infants required more days of invasive ventilation (median 64 vs. 47, *p* = 0.04) and more non-invasive respiratory support CPAP days (median 55 vs. 33, *p* < 0.01) than ELBW^501–750^ infants.

**Table 3:** Detailed respiratory and ventilator (first intention high frequency jet ventilation, HFJV) characteristics of ELBW^<501^ compared to ELBW^501–750^ infants from 2012 to 2017 (6).

	ELBW^<501^ *n* = 37	ELBW^501–750^ *N* = 112	*p*-value
Initial 2.0 mm ETT *n*, (%)	33, 89%	36, 32%	**<0**.**01**
Initial 2.5 mm ETT *n*, (%)	4, 11%	74, 66%	**<0**.**01**
Initial respiratory support, (%)			
Jet ventilation	37, 100%	106, 95%	0.15
CV	0%	1, 0.9%	0.56
CPAP	0%	5, 4%	0.19
Respiratory status at 6 h of age			
FiO_2_ (median, IQR)	0.33, 0.12	0.25, 0.14	**<0**.**01**
Mean airway pressure (cmH_2_O) (median, IQR)	8, 1	8, 1	0.08
Jet PIP (cmH_2_O) (median, IQR)	26, 8.5	23, 9.5	0.14
Jet rate BPM (median, IQR)	360, 60	360, 0	0.26
PCO_2_ at 6–12 h (mmHg)	44, 12	42,7	0.26
Respiratory status at 7 days of age			
FiO_2_ (median, IQR)	0.37, 0.12	0.32, 0.11	0.08
Mean airway pressure (cmH_2_O) (median, IQR)	8, 2	7, 2	0.13
Jet PIP (cmH_2_O) (median, IQR)	30, 10	26, 9	**<0**.**01**
Jet rate BPM (median, IQR)	300, 60	360, 60	0.72
PCO_2_ (mmHg)	52, 8.5	53, 8.25	0.62
Respiratory status at extubation			
FiO_2_ (median, IQR)	0.33, 0.09	0.32, 0.12	0.88
Mean airway pressure (cmH_2_O) (median, IQR)	10, 3	10, 3	0.06
Jet PIP (cmH_2_O) (median, IQR)	23, 3.5	21, 5	0.14
Jet rate BPM (median, IQR)	420, 60	390, 105	0.06
CV PIP (cmH_2_O) (median, IQR)	18, 7.5	18, 3	0.93
CV rate BPM	10, 2.5	10, 5	0.73
PCO_2_ (mmHg)	51, 9	50, 8	0.36
Extubation			
Age at first attempt (D) (median, IQR)	56, 30	44, 24	**<0**.**01**
PMA at first attempt (w), (median, IQR)	31.6, 2.8	30.6, 2.8	**0**.**049**
Age at final attempt (D) (median, IQR)	8.5, 17.8	50.5, 32	**<0**.**01**
PMA at final attempt (w), (median, IQR)	33.4, 2.8	32.1, 3.9	0.12
Total invasive ventilation days (median, IQR)	64, 27	47, 36	**0**.**04**
Total Nasal CPAP days (median, IQR)	55, 46	33, 20	**<0**.**01**

ETT, endotracheal tube; PIP, peak inspiratory pressure; CV, conventional ventilation; BPM, breaths per minute; CPAP, continuous positive airway pressure; W, week; D, days; PMA, post menstrual age.; IQR, interquartile range; ELBW, extremely low birth weight.

Bold values represent statistical significance.

## Discussion

In our 11-year cohort study of inborn infants with birthweight ≤750 g, stratified by birthweight, survival was significantly lower for ELBW^<501^ compared to ELBW^501–750^ infants. The lower survival rate in the ELBW^<501^ cohort was expected due to these infants being born at significantly lower gestational ages (23.2 weeks vs. 24.3 weeks, *p* < 0.01). However, the survival rate of ELBW^<501^ infants in our cohort, at 61%, was higher than recently reported rates of survival for infants born at ≤500 g (21%–55%) (13) but not as high as survival rates reported in a small Japanese case series (*n* = 10 live born infants) which demonstrated 80% survival for infants born at ≤500 g with comparable birthweights, though the Japanese infants were 2 weeks more mature (median BW of 436 g, median GA of 25.2 weeks) (14).

The high rate of survival in our ELBW^<501^ cohort was in part due to a strong commitment to proactive prenatal and neonatal care provided as previously described by Kyser et al. (15). Provision of at least one dose of ANS was generally high in both groups, however it is notable that significantly fewer infants in the ELBW^<501^ group received ANS which would have impacted survival. All the infants in this study received active neonatal care, including frequent use of 2.0 mm endotracheal tube (89% of ELBW^<501^ infants and 32% of ELBW^501–750^ infants). While 2.0 mm ETTs in themselves are not advantageous compared to 2.5 mm ETT, they are a marker of the provision of active resuscitation efforts for infants <501 g as there exists in some centers, a belief that if an infant requires a 2.0 mm ETT than concurrently they must be too developmentally immature to survive (6). Previous studies have shown improved survival for infants who received antenatal corticosteroids and were delivered by cesarean section, both markers of proactive prenatal care (13, 16–19). The larger ELBW^501–750^ infants were found to be more likely to be delivered via cesarian section and received ANS, which may have affected survival. At the University of Iowa, antenatal steroids are given to infants starting at 21 5/7th weeks gestation, however electronic fetal monitoring and cesarean section for fetal distress is not offered until 23 0/7 weeks. There was no signifcant difference between the groups in the use of post-natal steroids for chronic lung disease or surfactant use. ELBW^<501^ infants' length of stay was significantly more likely to exceed predicted discharge dates in comparison to ELBW^501–750^ infants based on GA alone. Our data support the importance of anticipatory guidance regarding increased LOS for families with infants born less than 500 g. The significant difference in discharge weight less than the 10th percentile between the two groups (*p* = 0.04) indicates that growth in this population remains a challenge and highlights the relevance of comprehensive nutritional protocols.

There were significantly more SGA infants in the ELBW^<501^ group compared to ELBW^501–750^ group (68.2% vs. 16.5%, *p* < 0.01). Small for gestational age is often used as a surrogate marker for fetal growth restriction and has been associated with increased mortality and adverse neonatal outcomes. The risk of neonatal death has been shown to be threefold higher in premature SGA compared to appropriate for gestational age (AGA) infants (13, 20–23) with higher risk of mortality at lower gestational ages and with more severe growth restriction (24, 25). In addition, death tends to occur earlier in SGA premature infants occurring in the delivery room or within the first 12 h of life (13). Because of this, infants born extremely premature and SGA are more often offered comfort care only over aggressive medical management (22). The true effect of SGA on the rate of common neonatal morbidities (NEC, ROP, IVH, sepsis, RDS, BPD, etc.) is unknown due to conflicting results reported in the literature (20–22, 24, 25).

However, studies consistently show that SGA infants have significantly longer hospital stays often due to increased severity of illness and significant neonatal morbidities (13, 24, 25). SGA infants are more likely to have received prenatal care, antenatal steroids, and be delivered by cesarean section, especially due to non-reassuring fetal heart rate tracing (20, 22). In addition, SGA infants seem to be at higher risk for slow weight gain, postnatal growth failure, prolonged ventilator support, postnatal steroid use, and chronic lung disease (13, 20, 22).

The initial respiratory approach for extremely premature infants with birthweights of ≤750 g that need invasive ventilation at the University of Iowa is to provide first intention high frequency jet ventilation using the Bunnell Life Pulse high frequency jet ventilator (Bunnell Incorporated Salt Lake City, UT). All ELBW^<501^ infants and 95% of the ELBW^501–750^ were placed on HFJV. HFJV management focused on minimizing mechanical injury from shear force through use of low tidal volumes (below physiologic dead space to reduce volutrauma), avoiding air trapping and hyperinflation by utilizing initial rates of 300–360 BPM with a fixed inspiratory time of 0.02 s to provide adequate time for exhalation. The use of this prolonged I:E ratio allows for adequate expiratory time for ventilation to occur by passive elastic recoil. A conventional ventilator runs in tandem to provide positive end expiratory pressure (PEEP) to maintain functional residual capacity and minimize atelectasis leading to loss of oxygenation. On admission no conventional sigh breaths are used with HFJV, however they are often added in the first week of life for alveolar recruitment to treat wandering atelectasis. Once conventional sigh breaths are added they are maintained until extubation to minimize recurrence of positional (wandering) atelectasis unless air leak develops (2, 3, 26). Sigh breaths are not used to regulate PCO_2_ but are used for desaturation spells associated with central apnea. HFJV rate may be increased (in 60 BPM increments) to optimize alveolar ventilation when the patient is having worsening hypercarbia despite increased PIP, and/or to improve oxygenation through increased lung recruitment as mean airway pressure rises with higher rates. Conversely, HFJV rate may be decreased for persistent hypocarbia despite weaning the Jet PIP. Air trapping [pulmonary interstitial emphysema (PIE) or hyperinflation] should be screened for with frequent radiographs, especially in the first week of life. If either PIE or hyperinflation is detected, the jet rate and MAP needs to be decreased and sigh breaths discontinued (2, 3, 26). First intention HFJV was recently found to reduce Grade 3 BPD in infants ≤26 weeks gestation (27).

In our study, the ventilator settings (respiratory parameter cohort 2012–2017) at 6 h of life were not significantly different based on birthweights except for the need for higher FiO_2_ in ELBW^<501^ infants compared to ELBW^501–750^ infants, consistent with their younger gestational age and less mature lungs. At 7 days of life, the PIP was significantly higher in the ELBW^<501^ than ELBW^501–750^ infants again likely related to lower gestational age and the use of a 2.0 mm ETT which requires higher PIP to overcome the increased resistance through the smaller ETT to deliver a similar tidal volume. PCO2 levels are followed closely and changes in the PIP or ΔP (PIP-PEEP) are adjusted per unit protocol to keep PCO2 levels 45–55 mmHg in the first week of life and then liberalized to 50–65 mmHg following stabilization of the germinal matrix. Due to strict regulation of PCO2 targets, there were no significant differences in levels between the two weight groups.

The chronic ventilatory approach for all ventilated infants with birthweights ≤750 g is to prioritize growth with stabilization of oxygenation and ventilation and to treat any hemodynamically significant PDA in the first 2 weeks of life before evaluation for extubation (28). Extubation failure in the first 2 weeks of life for infants <26 weeks gestation should be minimized since it is associated with a 5-fold increase in mortality, higher rate of BPD, severe IVH, and sepsis (29). The criteria for extubation from HFJV include strong sustained spontaneous respiratory drive, mean air way pressure ≤10–12 cm H_2_O, FiO_2_ ≤0.45, and delta P (PIP-PEEP) <14–16 cm H_2_O. In our study, the median age of successful extubation for ELBW^<501^ was 69 days compared with 51 days of life for ELBW^501–750^ infants (*p* < 0.01). It is important to emphasis that this study looked at two specific cohorts of ELBW infants with birthweights between 501 and 750 g and birthweights less than 501 g, rather than all ELBW or VLBW infants. It is likely that larger ELBW infants, particularly those closer to 1,000 g, will not require the same duration of mechanical ventilation. As expected, PMA at time of final extubation was not significantly different with a median of 33.4 weeks for the ELBW^<501^ infants and 32.1 weeks for the ELBW^501–750^ infants. However, due to ELBW^<501^ infants being more premature, they spent significantly more days on invasive ventilation (64 days vs. 47 days, *p* = 0.04) to reach the same level of lung maturation. Both groups of infants were extubated at similar MAP, PIPs, and FiO_2_ (*p* = 0.059, *p* = 0.14, and *p* = 0.88 respectively) consistent with our standardized respiratory guidelines. Infants were primarily extubated to non-invasive Neurally Adjusted Ventilatory Assist (NIV-NAVA) through a nasal pharyngeal tube (2, 3, 26). Per unit protocol, infants generally need to be >850–900 g to attempt extubation to NIV-NAVA. If below this weight and ready for a trial of extubation, NIPPV can be used.

While the need for supplemental oxygen at 36 weeks PMA was high in both groups (100% vs. 95.2% in ELBW^<501^ infants and ELBW^501–750^ infants) this is not unexpected and is consistent with work published involving infants born at similar gestational ages (11). Importantly, the incidence of Grade 3 BPD, which is associated with a two-fold higher rate of late death, serious respiratory morbidity, and moderate-to-severe neurodevelopmental impairment, was low in both groups and not significantly different. In addition, only 5.8% of the ELBW^<501^ infants and 3% of the ELBW^501–750^ infants required tracheostomy for ongoing mechanical ventilation at home (*p* = 0.23).

**Figure 2:**
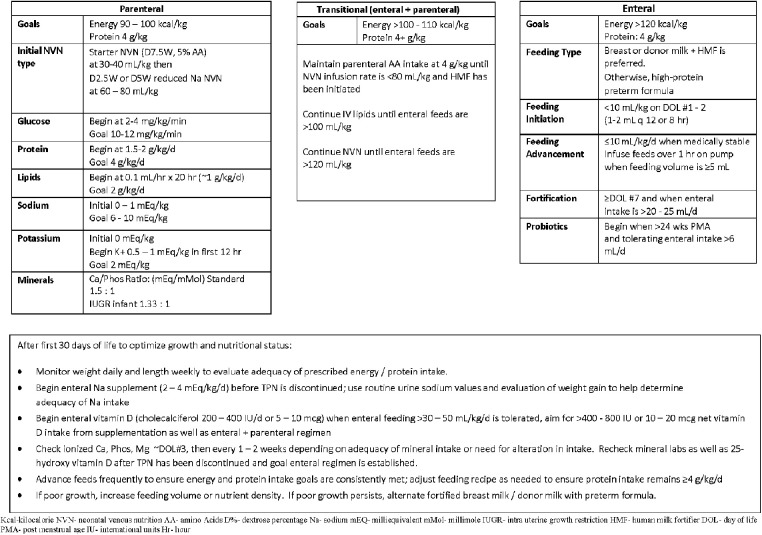
Feeding guidelines for infants born 22–23 weeks gestation or birth weight <501g.

Differences and similarities were seen in non-respiratory co-morbidities between the two groups. No significant differences were seen in PVL, NEC, or infection. Infants were fed according to comprehensive feeding guidelines. The feeding protocol for infants born at 22–23 weeks involves trophic feeds at 10 ml/kg initiated within the first 24–36 h with maternal breast milk or donor milk with slow advancements of 10–12 ml/kg/day if tolerating feeds based on residuals and physical exam. Bolus feeds are switched from straight gravity to pump over 1 h when absolute volume is >4 ml, and fortification is introduced when the infants are on day of life 7 and oral intake is greater than 20–25 ml/day (Figure 2). Close attention is paid to stooling patterns, if no stooling occurs after 2–3 days the infants receive glycerin suppositories. Feeds will not advance beyond 30 ml/kg/day in the first week until transitional stools are present. By the second week of life, if no transitional stools, or not tolerating trophic feeds or exam and x-rays are consistent with suspicion for meconium obstruction of prematurity, infants receive a contrast enema performed in the NICU. The expected time of full enteral feeds is 28–30 days of life.

The rates of any ROP and severe ROP were significantly higher among ELBW^<501^ infant survivors compared to the ELBW^501–750^ infants. Additionally, ELBW^<501^ infants were more likely to have the composite of ROP laser surgery and/or Anti-VEGF therapy compared to ELBW^501–750^ infants (*p* < 0.01). Increased risk for ROP in ELBW^<501^ infants is likely related to the degree of immaturity, and greatly increased number of SGA infants. The increase in admission hypothermia in the ELBW^<501^ infant is consistent with the inherent physiological challenges in maintaining euthermia in the population of micro- premature infants. Maintaining neonatal euthermia is critical, and decreased admission temperature is associated with NEC, ROP, BPD, sepsis and increased mortality (30–33). Policy for infants born less than 750 g is focused on resuscitation and stabilization then admission to the NICU within the first 15 min of life. The first temperature obtained is at the time of admission, and no pre-warming or temperature probes are used prior to reaching the NICU, though these interventions have been found to be successful in thermoregulation at other centers (34). Our center engages in robust thermoregulation simulation training focused on ELBW infants, and has seen improvement in our admission temperatures, but not at the extremes of birthweight and gestation age (35). Providing effective temperature management for the smallest of infants remains an ongoing challenge.

This study has several strengths and limitations. It provides detailed information about interventions, management strategies, survival, and short-term outcomes from a subset of high-risk preterm infants born less the 501 g. Additionally, as a single center study, all infants were treated similarly based on a single institution's practices and policies. We acknowledge that our study has several limitations including a retrospective cohort design from a large database which led to the inability to obtain individualized ventilator data beyond the earlier detailed ventilator study from 2012 to 2017. Additionally, the database does not include neurodevelopmental outcomes at 18–22 months of corrected age, however we would not suspect it to be different then what was found in our previous cohort of infants born between 2006 and 2015 at 22–25 weeks gestation. It will be important to gather follow up data at 2 years corrected age in this new cohort of infants born from 2016 to 2022 to examine long term neurodevelopmental outcomes for the <501 g population compared to the 501–750 g group.

In conclusion, ELBW^<501^ infants are at risk for significant mortality compared to the ELBW^501–750^ infants. However, with proactive obstetrical care and specialized neonatal care including the use of 2.0 mm endotracheal tubes when required and first intention high frequency jet ventilation, survival rates of 61% are possible. Although 100% of these infants had BPD defined as supplemental oxygen at 36 weeks PMA, rates of grade 3 BPD (need for invasive ventilation at 36 weeks PMA) and tracheostomy were very low with the use of first intention HFJV and importantly, rates of IVH, PVL and NEC were not higher in the smaller group. ELBW^<501^ infants as expected have a longer hospital stay with more days of invasive ventilation and are at a much higher risk of ROP, and thus families should be counseled appropriately. With the exception of ROP (and ROP requiring intervention), this vulnerable patient population (<501 g) has comparable morbidities which may potentially impact long term neurodevelopmental outcomes. Thus, it will be important to continue to obtain and report on long term follow-up for this high-risk population.

## Data Availability

The raw data supporting the conclusions of this article will be made available by the authors, without undue reservation.
